# Elevated C-reactive protein values predict nodal metastasis in patients with penile cancer

**DOI:** 10.1186/1471-2490-13-53

**Published:** 2013-10-22

**Authors:** Andreas Al Ghazal, Sandra Steffens, Julie Steinestel, Rieke Lehmann, Thomas J Schnoeller, Anna Schulte-Hostede, Gerd Wegener, Florian Jentzmik, Mark Schrader, Markus A Kuczyk, Andres J Schrader

**Affiliations:** 1 Department of Urology, Ulm University Medical Center, Ulm, Germany; 2 Department of Urology, Hannover Medical School, Carl-Neuberg-Str. 1, D-30625 Hannover, Germany; 3 Cancer Center, Hannover Medical School, Hannover, Germany

**Keywords:** Penile cancer, Biomarker, C-reactive protein, Nodal disease, Prognosis, Survival

## Abstract

**Background:**

The nodal status is a strong predictor for cancer specific death in patients with penile carcinoma, and the C-reactive protein (CRP) level at diagnosis has recently been shown to be associated with poor clinical outcome in various solid malignancies. Therefore, this retrospective study was performed to evaluate the association between preoperative CRP levels and the incidence of nodal metastasis in patients with squamous cell carcinoma (SCC) of the penis.

**Methods:**

The analysis included 51 penile cancer patients who underwent either radical or partial penectomy for pT1-4 penile cancer between 1990 and 2010. The nodal status was correlated with patient and tumor specific characteristics.

**Results:**

Sixteen (31%) patients had lymph node metastasis at the time of penile cancer surgery. Nodal status was associated with tumor stage but did not correlate significantly with tumor grade. In contrast, high presurgical CRP levels were significantly associated with the diagnosis of nodal involvement (p = 0.04). The optimal CRP cut-off value to predict lymph node metastasis was set at 20 mg/l based on ROC analysis.

**Conclusions:**

Since a high preoperative serum CRP level was closely correlated with nodal disease, it could be used as an additional marker to help identify patients with penile cancer who may benefit from inguinal lymph node dissection.

## Background

Squamous cell carcinoma (SCC) of the penis, which accounts for >95% of penile cancer cases, is relatively uncommon in the Western world, but its incidence has increased slightly and varies markedly in some parts of Europe with an annual rate of 0.5 to 1.6 per 100,000 men. Its incidence in the United States is affected by race and ethnicity, being low for Asian-Pacific Islanders and American Indians and highest for Hispanics and Southern Blacks [1,2]. The most important prognostic factor in SCC of the penis is inguinal lymph node involvement [2,3]. Optimal management of patients with impalpable or even palpably enlarged nodes has been under debate for many years [4,5]. Various approaches advocated in the past include close surveillance, dynamic sentinel node biopsy, modified lymphadenectomy, and risk-adapted or elective lymphadenectomy [3,6]. Each approach has its advantages and disadvantages. Early resection definitely offers a survival benefit [2,7], but unfortunately, patients who undergo inguinal lymphadenectomy are prone to short- and long-term morbidity [8]. Although the therapeutic benefits outweigh these complications in patients with pathologic nodal involvement [9], only 20% of those with impalpable lymph nodes harbor occult metastasis [3,10]. Prophylactic inguinal lymph node dissection in all these patients would expose many of them to increased morbidity without offering much benefit. Moreover, up to 50% of all palpable inguinal nodes at diagnosis of penile cancer are caused by inflammatory reactions [2,11]. The high morbidity associated with inguinal lymph node dissection combined with a high rate of negative histopathological findings accounts for the fact that lymphadenectomy is not performed in many cases, even when indicated according to all current guidelines (higher risk factors, e.g. tumor stage ≥ pT1b) [12].

Classical molecular markers are of no clinical value in SCC of the penis. The soluble eptithelial antigen SCC lacks sensitivity in the detection of small tumor burdens and has little prognostic significance for survival after surgery [13]. The detection of lymph node metastases has been associated with the overexpression of p53 and Ki-67, as well as loss of membraneous E-cadherin, but these markers are not useful in clinical practice [2,14].

C-reactive protein (CRP) is an acute-phase reactant to microbial infection, trauma, infarction, autoimmune diseases or malignancies [15]. High levels of circulating CRP have recently been associated with metastatic disease and a poor prognosis in various malignancies [16-26], including renal cell carcinoma [27,28], urothelial carcinoma [29,30], castration-resistant prostate cancer [31], and even penile cancer [26].

This retrospective two-center study was initiated to evaluate the impact of CRP levels at diagnosis on lymph node metastasis in patients with penile cancer.

## Methods

### Patient and tumor characteristics

The 51 patients included in this study had complete patient and tumor characteristics, including the preoperative CRP level, and underwent surgery for penile cancer between 1990 and 2010 at the Ulm (n = 24) or Hannover (n = 27) University Medical Centers. The study was approved by the Ulm University ethics committee (proposal no. 241/12). All research has been carried out according to the current Helsinki Declaration (59^th^ edition, Seoul, Korea, 2008; http://www.wma.net/en/30publications/10policies/b3). The histological tumor subtype was determined according to the 2010 UICC Classification. Patient and tumor characteristics obtained from our computerized institutional databases included age, stage, regional lymph node involvement or distant metastasis, histological subtype, tumor grade, CRP value, and body mass index (BMI) as well as rural vs. urban residential area. The median (mean) follow-up was 27 (37) months; follow up data were evaluable for n = 49 patients.

### Statistical methods

Continuous variables were reported as the mean and the interquartile range (IQR). Chi-square and Fisher’s exact tests were conducted to assess correlations between the distribution of covariates and nodal involvement. Mann–Whitney tests were applied to compare continuous cardinal parameters between the two groups, i.e. patients with and without lymph node metastasis.

Receiver operating characteristic (ROC) curves were plotted to assess the potential of preoperative CRP for predicting lymph node involvement.

SPSS 19.0 was used for statistical analysis. A two-tailed p value less than 0.05 was considered significant in all tests.

## Results

### Patient and tumor characteristics

Our patient population comprised 51 men with a mean age of 62.9 (33–88) years who suffered from SCC of the penis and underwent penile cancer surgery. 34 patients (67%) lived in rural and 17 (33%) in urban areas.

The total patient population had a mean body mass index (BMI) of 27.2 kg/m^2^ (IQR, 24.2 – 29.8 kg/m^2^) and a mean CRP value of 16.2 mg/l prior to surgery. Thus 25 (49%) patients presented with CRP values within the normal range (<5 mg/l), while 26 (51%) patients had an elevated CRP value at the time of penile surgery. Thirty-two of all patients (63%) suffered from locally advanced penile cancer (≥pT2); nodal disease was seen in 16 cases (31%) and distant metastasis in 4 cases (8%) at the time of surgery.

Table 1 gives a detailed summary of patient and tumor characteristics, including stage and grade.

**Table 1:** Association between different patient or tumor variables and nodal metastasis

**Variable**	**LN negative**	**LN positive**	**p-value**	**Test**
Age, mean [years] (IQR)^1^	62.5 (56.0-71.5)	63.8 (61.3-68.4)	0.9	Mann–Whitney
BMI, mean [kg/m^2^] (IQR)^1^	27.5 (24.1-30.3)	26.7 (26.1-28.4)	0.9	Mann–Whitney
Residential area			0.5	Fisher’s exact
Urban	12 (35%)	4 (25%)		
Rural	22 (65%)	12 (75%)		
Stage			0.01	Chi^2^
pT1	15 (43%)	4 (25%)		
pT2	15 (43%)	5 (31%)		
pT3	3 (9%)	5 (31%)		
pT4	2 (6%)	2 (13%)		
Pulmonal/visceral metastasis^1^			0.01	Fisher’s exact
M0	33 (100%)	12 (75%)		
M1	0	4 (25%)		
Grade			0.10	Chi^2^
G1	6 (18%)	1 (6%)		
G2	21 (62%)	8 (50%)		
G3/4	7 (21%)	7 (44%)		
CRP value, mean [mg/l], (IQR)^1^	12.4 (3.0-8.0)	24.7 (4.0-46.3)	0.04	Mann–Whitney

^1^at time of penile cancer surgery. *Abbreviations*: *CRP* C-reactive protein, *BMI* body mass index, *LN* lymph node, *SD* standard deviation.

### Association between lymph node involvement and patient or tumor characteristics

Lymph node metastasis did not correlate with age (p = 0.9, Mann–Whitney test) or BMI (p = 0.9, Mann–Whitney test) (cf. Table 1). Moreover, the nodal status was not statistically significantly related to the residential area (rural vs. urban; p = 0.5, Fisher’s exact test) or tumor grade (p = 0.1, chi^2^ test; Table 1). In contrast, the presence of lymph node involvement was significantly associated with tumor stage (p = 0.01, chi^2^ test). Only patients with nodal involvement had simultaneous visceral metastasis (p = 0.01, Fishers’ exact test).

In addition, the mean CRP value was significantly higher in patients with nodal disease than in those without it: 24.7 mg/dl vs. 12.4 mg/dl (p = 0.04, Mann–Whitney test).

Receiver operating characteristic (ROC) analysis showed that the AUC (95% CI) of the CRP value was 0.68 (0.51 – 0.85; p = 0.04) for lymph node metastasis at the time of penile surgery. Moreover, this analysis revealed an optimal CRP cut-off of 20 mg/l for predicting lymph node metastasis.

Twenty-four patients presented with palpable and clinically suspicious inguinal lymph nodes at the time of penile surgery. Ultimately, 16 (67%) of them had metastasis in the histological specimen after lymph node dissection. The mean CRP value was higher in patients with clinically suspicious and tumor-positive nodes than in those whose inguinal lymph nodes were clinically suspicious but eventually tumor negative (24.7 vs. 6.4 mg/l). However, this difference was not statistically significant (p = 0.07, Mann–Whitney test).

## Discussion

Elevated plasma CRP levels are not only associated with an increased risk of cancer but also have been linked to advanced disease with poor prognosis in various malignancies [16-30]. In a recently published study we were able to show that a high preoperative serum CRP level was associated with poor survival in patients with penile cancer [26]. In addition, this study shows that elevated CRP levels are associated with higher tumor stages and nodal disease, the most important prognostic factors for penile carcinoma.

Our results are in line with those obtained for other tumor entities. Chen et al. [16] found that high circulating CRP levels correlated significantly with lymph node metastasis and survival in patients with oral SCC. Ishizuka et al. [32] evaluated several potential clinical factors and biological markers in a large series of patients with locally advanced colorectal cancer. Multivariate analysis identified high CRP levels as an indicator or predictor of both nodal and distant metastasis in T3 colorectal cancer. Neuss et al. [21] demonstrated that the preoperative serum CRP level correlated significantly with the number of lymph node metastases found during radical lymph node dissection in stage III melanoma patients.

Approximately 50% of the palpable lymph nodes in patients with penile cancer ultimately prove to be tumor-free after inguinal lymphadenectomy. The reason for the low positive predictive value of palpable nodes is that their enlargement can also caused by reactive changes upon inflammatory reactions [2]. We therefore focused on the subgroup of our patients with palpable nodes (n = 24), all of whom underwent nodal dissection. Sixteen of them (67%) actually did have lymph node metastasis. The question was raised as to whether the CRP value might help to distinguish between inflammation and cancer spread as the cause of nodal enlargement. However, CRP level did not differ significantly between the two entities in this small subgroup, although it tended to be even higher in the patients with nodal metastasis (mean, 24.7 vs. 6.4 mg/l). This clearly indicates that the tumor itself seems to be involved in the elevation of serum CRP levels and that the phenomenon of elevated CRP in penile cancer is not simply a consequence of superinfection.

Our study was not without limitations. First and foremost among them is its retrospective design, which precluded the systematic evaluation of important additional prognostic factors such as microscopic lymphovascular and perineural invasion, growth pattern, and anatomic site. Moreover, superinfection of penile tumors was not documented thoroughly and thus, retrospectively, we could not assess this potentially confounding factor. The study is also limited by the lack of central pathologic review. Finally the number of included patients was relatively small.

## Conclusion

In conclusion, we have shown that a high preoperative serum CRP level is closely correlated with nodal disease and could help identify patients with penile cancer who may profit from inguinal lymph node dissection. Nomograms have already been proposed to estimate the cumulative risk of metastatic involvement in penile cancer patients, using different clinical and pathological parameters [33]. We will have to evaluate whether the predictive significance of these models can be further improved by including additional markers such as the preoperative serum CRP level.

## Competing interests

We declare that we have no conflict of competing interests.

## Authors’ contributions

AAG was part of the acquisition, analysis and interpretation of data and drafted the manuscript. SS designed and planed the study, was part of the acquisition, analysis and interpretation of data and contributed to the drafting of the manuscript. JS, RL, TJS, ASH, GW and FJ were part of the data acquisition. MS and MAK were responsible for the supervision. AJS planed the study, was part of the acquisition, analysis and interpretation of data, performed the statistical analysis and drafted the manuscript. All authors read and approved the final manuscript.

## Pre-publication history

The pre-publication history for this paper can be accessed here:

http://www.biomedcentral.com/1471-2490/13/53/prepub
